# Gut Hormones and Their Effect on Bone Metabolism. Potential Drug Therapies in Future Osteoporosis Treatment

**DOI:** 10.3389/fendo.2019.00075

**Published:** 2019-02-26

**Authors:** Sine Paasch Schiellerup, Kirsa Skov-Jeppesen, Johanne Agerlin Windeløv, Maria Saur Svane, Jens Juul Holst, Bolette Hartmann, Mette Marie Rosenkilde

**Affiliations:** ^1^Laboratory of Molecular Pharmacology, Department of Biomedical Sciences, Faculty of Health and Medical Sciences, University of Copenhagen, Copenhagen, Denmark; ^2^Department of Biomedical Sciences, Faculty of Health and Medical Sciences, University of Copenhagen, Copenhagen, Denmark; ^3^Faculty of Health and Medical Sciences, Novo Nordisk Foundation (NNF) Center for Basic Metabolic Research, University of Copenhagen, Copenhagen, Denmark

**Keywords:** gut hormones, bone metabolism, GIP, GLP-1, GLP-2, PYY, osteoporosis

## Abstract

Bone homeostasis displays a circadian rhythm with increased resorption during the night time as compared to day time, a difference that seems—at least partly—to be caused by food intake during the day. Thus, ingestion of a meal results in a decrease in bone resorption, but people suffering from short bowel syndrome lack this response. Gut hormones, released in response to a meal, contribute to this link between the gut and bone metabolism. The responsible hormones appear to include glucose-dependent insulinotropic polypeptide (GIP) and glucagon-like peptide-1 (GLP-1), known as incretin hormones due to their role in regulating glucose homeostasis by enhancing insulin release in response to food intake. They interact with their cognate receptors (GIPR and GLP-1R), which are both members of the class B G protein-coupled receptors (GPCRs), and already recognized as targets for treatment of metabolic diseases, such as type 2 diabetes mellitus (T2DM) and obesity. Glucagon-like peptide-2 (GLP-2), secreted concomitantly with GLP-1, acting via another class B receptor (GLP-2R), is also part of this gut-bone axis. Several studies, including human studies, have indicated that these three hormones inhibit bone resorption and, moreover, that GIP increases bone formation. Another hormone, peptide YY (PYY), is also secreted from the enteroendocrine L-cells (together with GLP-1 and GLP-2), and acts mainly via interaction with the class A GPCR NPY-R2. PYY is best known for its effect on appetite regulation, but recent studies have also shown an effect of PYY on bone metabolism. The aim of this review is to summarize the current knowledge of the actions of GIP, GLP-1, GLP-2, and PYY on bone metabolism, and to discuss future therapies targeting these receptors for the treatment of osteoporosis.

## Introduction—Bones

Bone is a tissue with very important mechanical functions, providing rigidity, strength and shape, and is essential for movement. However, in spite of its apparently static structure, bone tissue is dynamic and undergoes a constant remodeling, consisting of processes of bone resorption and bone formation. Proper balance is controlled by the coupling of these two processes, and involves a number of coordinated signaling mechanisms. In normal bone remodeling, a balance between bone resorption (mediated by osteoclasts) and bone formation (mediated by osteoblasts) is maintained to ensure a constant bone mass. An imbalance between bone resorption and bone formation may occur under certain pathological conditions, and lead to abnormal bone remodeling and the development of bone disorders (1). Bone also has an important function as a reservoir for calcium and phosphate, bound in the matrix as hydroxyapatite, and bone tissue is, therefore, along with the intestine and kidneys, important for the maintenance of proper calcium levels (2–4).

Histologically, there are two main types of bone, cortical and trabecular. These have different structure and properties. Cortical bone has a highly organized, lamellar structure, providing planar strength. Generally, bones have an outer layer of cortical bone with trabecular bone beneath. The weight-bearing long bones, such as the femur and humerus, mainly have cortical bone in their shafts. Trabecular bone has a more irregular and less dense structure, consisting of interconnecting bars, or trabeculae, with bone marrow filling the gaps. The number of trabeculae has been shown to be more important than their thickness in regard to the strength of the bone (5).

Bone tissue consists of cellular elements within an extracellular matrix. The cellular components are the osteoblasts, the osteocytes and the osteoclasts. The bone-forming osteoblasts derive from mesenchymal stem cells that differentiate into osteoprogenitor cells, a process that is dependent on the Wnt/β-catenin pathway. With age, the osteoblasts become buried in the matrix and are now designated osteocytes. These cells communicate with each other and with other cells, particularly those on the surface of the bone tissue, through dendritic processes in canaliculi in the bone, allowing them to regulate bone-turnover in response to mechanical stress (1). Osteoclasts are multinucleated cells, derived from the macrophage/monocyte lineage, which resorb bone on the growth surface. The differentiation into mature osteoclasts is dependent on activation by the receptor activator of nuclear factor κB (NF-κB) ligand (RANKL) and monocyte colony stimulating factor (M-CSF) produced by the osteoblasts.

**Figure 1:**
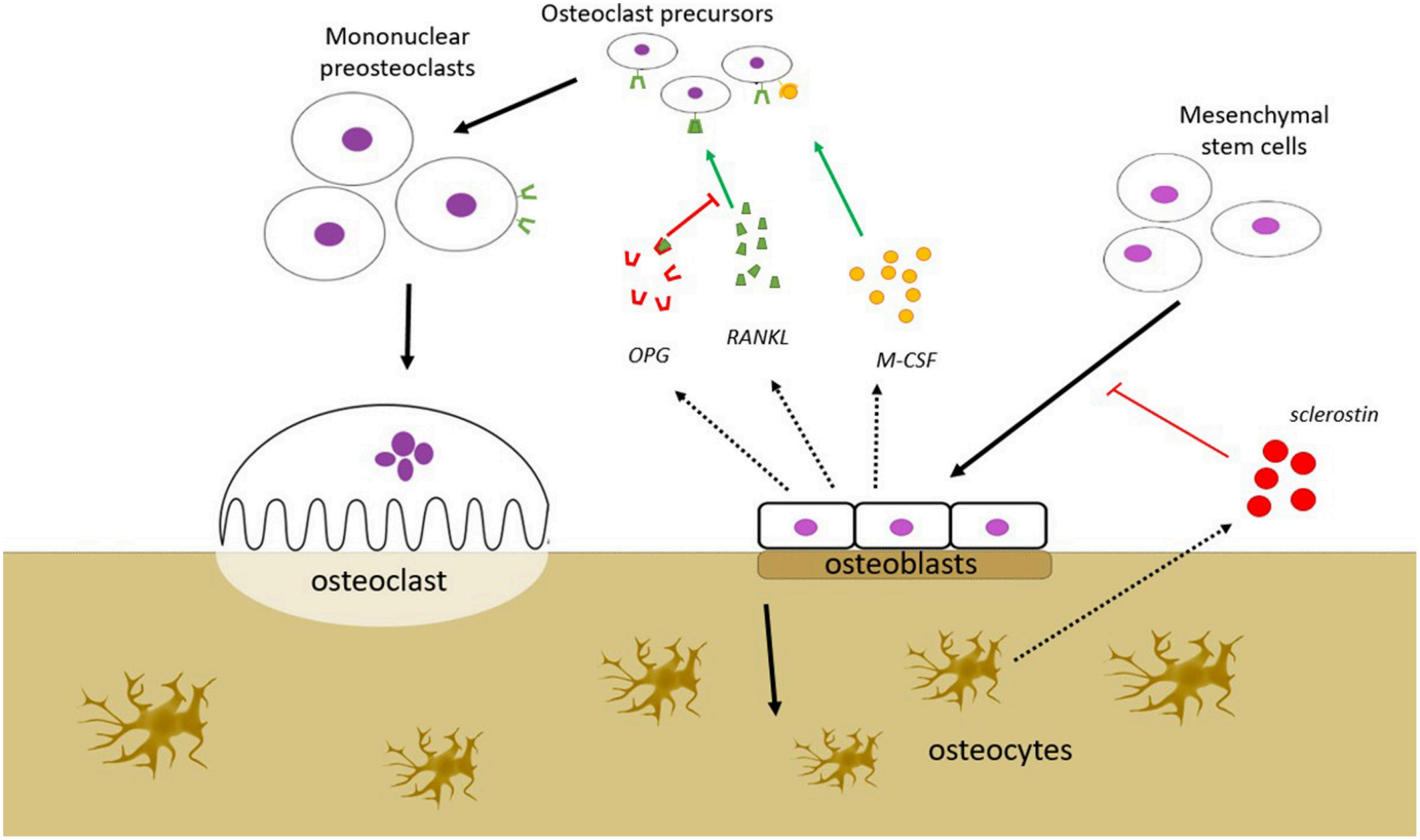
Bone remodeling is a process that takes place in the bone remodeling unit (BMU). BMU consists of bone resorbing osteoclasts (OC), bone forming osteoblasts (OB), and osteocytes. The process is regulated by local signals between the cells and by external stimuli. OBs stimulate OC precursors to differentiate into mature OCs by secreting monocyte colony stimulating factor (M-CSF) and RANKL, but may also inhibit the same cells by secreting osteoprotegerin (OPG) that scavenges RANKL, preventing it from binding to the RANK receptors on the OC precursors. OBs are derived from mesenchymal stem cells, a process that is dependent on the Wnt/β-catenin pathway. This pathway is inhibited by sclerostin, which is secreted from osteocytes. Several other factors which affect bone remodeling are mentioned in the text (FGF-23, BMP), but are not shown in this figure.

The remodeling of the bone involves a coordinated action of a team of cells, referred to as a basic multicellular unit (BMU). The osteoclast-mediated resorption and the osteoblast-mediated formation are thought to be orchestrated by local regulation within the BMU, as well as by systemic regulation from outside the BMU. The local signaling within the BMU is often presented as a complex network of regulation between the different cell types, where the osteocyte population regulates the activity of the osteoblast population, and the osteoblast population, in turn, regulates the activity of the osteoclast population and vice versa (Figure 1). Osteocytes regulate the osteoblasts via signaling molecules including fibroblast growth factor-23 (FGF-23), bone morphogenetic proteins (BMPs), and sclerostin, whereas some of the key signaling molecules involved in the osteoblastic regulation of the osteoclasts include RANKL, that induces osteoclast activity, and osteoprotegerin (OPG), that counters the effect of RANKL. Malfunctions in some of these regulatory mechanisms are known to cause bone disorders; mutations of FGF-23, for example, are known to cause autosomal dominant hypophosphatemic rickets (ADHR). Some of these regulatory factors, including RANKL and sclerostin, are targets for therapies that aim to alter the balance between the osteoblast activity and osteoclast activity in order to treat osteoporosis, which is characterized by an imbalance between these activities.

When bone is resorbed, a carboxy-terminal telopeptide of type I collagen (CTX) is liberated and released into the blood stream. Levels of circulating CTX are, therefore, used as a biomarker for bone resorption. CTX levels show diurnal variation, peaking during the night-time, and showing a nadir in the late afternoon. As fasting is associated with a flattened circadian rhythm (6), with the day-time decline in CTX levels being eliminated, ingestion of food seems to be the explanation for the daytime suppression (7, 8). Bone formation can be assessed by measurements of an amino-terminal propeptide of type 1 procollagen (P1NP) or by measuring the protein osteocalcin, which is secreted from osteoblasts.

## The Gut-Bone Axis

The gut and the bones are connected through the gut-bone axis, and this interaction is mediated by hormones secreted from the intestine. These hormones are secreted in response to food intake and causes a decrease in bone resorption (7–9), and are thus mediators of the adaptation of bones to nutrient availability. Bone resorption is increased during the night as compared to the day, and this day-time suppression is eliminated by fasting, further affirming the role of the gastrointestinal hormones in the control of bone homeostasis. Many hormones are secreted from the gut [for a recent review see Gribble and Reimann (10)], but the focus of this review will be on the incretin hormones glucose-dependent insulinotropic polypeptide (GIP) and glucagon-like peptide-1 (GLP-1), as well as glucagon-like peptide-2 (GLP-2) and peptide YY (PYY).

GIP (secreted from enteroendocrine K-cells) and GLP-1 (secreted from L-cells) have been extensively studied with respect to their effects on glucose metabolism as mediators of the incretin effect: i.e., the enhanced insulin secretion which occurs when glucose is ingested orally compared to i.v. glucose injection (11–14). For this reason, there has been much interest in their use in the treatment of type 2 diabetes mellitus (T2DM) and obesity, and many widely used drugs for T2DM, such as liraglutide and exenatide, act as GLP-1 receptor agonists (GLP-1RA). GLP-2 is also released from the L-cells in the small intestine, but in contrast to the glucose-lowering effects of GLP-1 (and GIP), GLP-2 is mostly known as an intestinotropic factor (15, 16). GIP, GLP-1, and GLP-2 are all believed to protect against bone resorption, either via direct effects on the bone cells, or indirectly. PYY is co-secreted with GLP-1 and −2 from the L-cells and also affects bone metabolism, possibly by inhibiting formation. These hormones are involved in the gut-bone axis, as reviewed below and summarized in Table 1.

**Table 1:** Summary of the known effects of the gut hormones GLP-2, GIP, GLP-1, and PYY on bone metabolism.

**Hormone**	**Study design**	**Species**	**Finding**	**References**
GLP-2	*In vivo*	Human	GLP-2 inhibits bone resorption (measured as CTX) with only minimal effects on bone formation (measured as osteocalcin or P1NP). Four months of GLP-2 treatment increases hip BMD in postmenopausal women	(7, 17–19)
		Mouse	None. No studies report effects of GLP-2 on bone remodeling in the mouse	
		Rat	None	
	*In vitro*	Human	The GLP-2R has not been identified in human bone cells, though one study reports GLP-2R expression on the cell lines MG-63 and TE-85 (reflecting immature human osteoblasts)	(20)
		Mouse	None	
		Rat	None	
Summary GLP-2: GLP-2 does not affect bone remodeling in rodents either *In vitro* or *In vivo*. In humans, GLP-2 acutely inhibits bone resorption, and 4 months of GLP-2 treatment increases hip BMD in postmenopausal women				
GIP	*In vivo*	Human	GIP reduces CTX independently of insulin Loss-of function GIPR gene polymorphism is correlated to a decreased BMD and increased fracture risk	(9, 21–23)
		Mouse	Lack of GIP signaling or peptide alters the bone structure in a negative direction, but findings in different studies are not always consistent	(24–27)
		Rat	GIP Improves bone density in OVX rats and cortical bone properties	(28, 29)
	*In vitro*	Human	GIP reduces osteoclast formation and resorption In osteoblastic cell lines, GIP increase stimulates ALP and PINP, and diminishes cell death	(20, 30–32)
		Mouse	GIP inhibits PTH induced resorption and stimulates ALP and mineralization in osteoblasts	(33, 34)
		Rat	None	
Summary GIP: GIP has a direct effect on regulation on bone metabolism with anabolic effects on osteoblasts and anti-resorptive effects on osteoclasts				
GLP-1	*In vivo*	Human	GLP-1 has positive effects on bone metabolism, possibly through increased formation. No effect on plasma CTX concentrations	(7, 22, 35)
		Mouse	Studies in mice show that treatment with GLP-1RAs have protective effects against OVX-induced or diabetes-induced bone loss	(36, 37)
		Rat	GLP-1 has positive effects on bone strength and quality, and protects against bone loss. It causes an increase in bone formation parameters and a decrease in bone resorption parameters	(38–44)
	*In vitro*	Human	The receptor has been found on some osteoblastic cell lines. GLP-1 also increases cell viability and promotes osteogenic differentiation of BMSCs	(20, 38, 40, 45)
		Mouse	The receptor has been found in mouse osteoblast-like cells. In most studies, GLP-1 leads to an increase in differentiation and proliferation of osteoblasts, and it also exerts effects on osteoclasts	(37, 46–49)
		Rat	The GLP-1R has been found on rat osteoblasts and osteocytes, and GLP-1 affects the osteoblastic differentiation and regulate osteocyte protein production	(39, 40)
Summary GLP-1: GLP-1 directly affect bone cells, and regulates bone turnover by increasing formation and decreasing resorption				
PYY	*In vivo*	Human	Inverse relationship between plasma PYY and BMD in populations with weight gain (↓PYY and ↑BMD in obesity) and weight loss (↑PYY and ↓BMD in patients with anorexia and after gastric bypass surgery)	(50–52)
		Mouse	Direct effect of PYY on osteoblast and osteoclast activity with a negative relationship between PYY and osteoclast activity PYY^−/−^ mice exhibit an increase in bone mass and strength, although controversies exist Regulation of bone resorption and formation seem to occur via Y1, Y2, and Y6 receptors	(53–57)
		Rat	None	
	*In vitro*	Human	None	
		Mouse	PYY signaling in osteoblasts occurs via Y1 receptors, but no Y receptors seem to exist on osteoclasts	(54)
		Rat	None	
Summary PYY: PYY may play a role in bone mass regulation as evident from association studies in populations with altered energy balance. Support of this originates from rodent studies				

### Glucagon-Like Peptide-2 (GLP-2)

GLP-2 is co-secreted with GLP-1 from intestinal L-cells in the small and large intestine upon nutrient ingestion. GLP-1 and GLP-2 are derived from pro-glucagon which is post-translationally processed by pro-hormone convertase 1/3 in the L-cells (58–60). Intact GLP-2(1-33) (referred to as GLP-2 in rest of this review) is cleaved by the ubiquitous protease dipeptidyl peptidase-4 (DPP-4) at the alanine in position 2, with a half-life in plasma of approximately 7 min, forming the main degradation product GLP-2(3-33) (61). This variant has been shown to act as a low affinity, partial agonist with competitive antagonistic properties on the GLP-2 receptor *in vitro* (62). A prolonged half-life of GLP-2 can be achieved by substitution of the alanine in position 2 or by the use of DPP-4 inhibitors (63–65).

The GLP-2 receptor (GLP-2R) belongs to class B G protein-coupled receptors (GPCR). Based on animal studies, it is predominantly expressed in the gastrointestinal tract in enteric neurons (66, 67), but has also been found in the central nervous system and may be sparsely expressed in the lungs (20, 66). The exact localization of the GLP-2R in humans is however still uncertain due to lack of good antibodies for immuno-localization, as well as the low levels of GLP-2R expression in cells outside the gastrointestinal tract; extrapolation from other species may be risky because of differences between species (68).

GLP-2 has trophic effects on the intestine. In mice, administration of GLP-2 promotes growth of the small and large intestine, whereas co-administration of GLP-2 and high doses of GLP-2(3-33) results in a reduced response (62). GLP-2 acts on the intestinal crypt compartment, stimulating proliferation, but also inhibits apoptosis (69). Accordingly, GLP-2 has been studied in patients with short bowel syndrome (SBS) (70) and, since 2012, a DPP-4 resistant GLP-2 analog (teduglutide) has been used in the treatment of SBS. GLP-2 also appears to improve the intestinal barrier function, up-regulate glucose transport and increase mesenteric blood flow. Although less well-established, it has also been reported to inhibit food intake and promote neuronal proliferation (17, 18, 71, 72). The mechanisms underlying the effects of GLP-2 are not well-described, although they seem to be mediated indirectly through the ErbB system (73), keratinocyte growth factor (KGF) (74) and, perhaps, insulin-like growth factor-1 (IGF-1) (69).

#### GLP-2's Effect on Bones

In 2001, the first human study revealed that 5-weeks treatment with natural GLP-2 significantly increased spinal areal bone mineral density (aBMD) in SBS patients with no terminal ileum and no colon (70, 75). Shortly hereafter, Henriksen et al. showed that GLP-2 administered subcutaneously (s.c.) in doses ranging from 200, 400, and 800 μg in healthy postmenopausal women dose-dependently reduced bone resorption (measured as CTX), while the bone formation (measured as osteocalcin) was unaffected (7). Moreover, GLP-2 s.c. injected at bedtime also inhibited the nocturnal bone resorption (CTX) (76). In a 14-day study, daily bedtime injections of 1.6 and 3.2 mg GLP-2 were well-tolerated and reduced CTX with no effect on markers of bone formation (osteocalcin and P1NP) (19). Finally, 4 months of GLP-2 treatment resulted in a dose-related significant increase in hip aBMD of about 1%, with no signs of GLP-2 antibodies or tachyphylaxis (17). In contrast to the earlier findings that GLP-2 increased aBMD in SBS patients (75), Gottschalck et al. reported that reductions in CTX after exogenous GLP-2 requires an intact small intestine, indicating an indirect effect of GLP-2 involving the intestine (77, 78). Additionally, they found that GLP-2 decreased PTH in control participants with an intact intestine, making PTH a potential mediator of the GLP-2 induced decrease in CTX (77). In 2013, Askov-Hansen et al. investigated the effect of high concentration (achieved by i.v. injection) vs. prolonged exposure (achieved by s.c. injection) of GLP-2 in healthy participants. They found that prolonged exposure was more effective in reducing circulating CTX levels than acute high concentrations. Pre-treatment with the DPP-4 inhibitor sitagliptin increased plasma levels of GLP-2, but had no additional effects on CTX (18). Intriguingly, despite the impact of GLP-2 on osteoclast activity, the GLP-2R has not been identified in human osteoclasts, or in any other bone-related cell type, though Pacheco-Pantoja et al. found the receptor to be expressed in the immature human osteoblast cell lines MG-63 and TE-85 (20).

In summary, GLP-2 markedly inhibits bone resorption with only minimal effects on bone formation, resulting in an increased bone mineral density. Judged from existing studies, only supra-physiological doses of exogenous GLP-2 reduce bone resorption (CTX), but the mechanism by which GLP-2 affects bone metabolism is still unknown. It might act directly on bone cells or the effect might be mediated indirectly, possibly involving other intestinal factors.

### Glucose-Dependent Insulinotropic Polypeptide (GIP)

GIP is a 42 amino acid peptide secreted upon food ingestion from the enteroendocrine K-cells located primarily in the proximal small intestine (71). Together with GLP-1, it is known as an incretin hormone, being responsible for approximately 50–70% of the insulin response to oral glucose administration in healthy humans (71). GIP(1-42) is, like GLP-2, N-terminally cleaved by DPP-4 generating the metabolite GIP(3-42) (79), thereby resulting in a plasma half-life of active GIP of 4 min in humans (80). For research purposes, several DPP-4 resistant GIP analogs have been produced, such as N-AcGIP, Pro^3^GIP, and D-Ala_2_-GIP. Moreover, a naturally occurring C-terminally truncated variant, devoid of the last 12 amino acids, GIP(1-30)NH_2_, acts as full agonist for the human GIP system (81). DPP-4 cleavage of this compound results in GIP(3-30)NH_2_, a high affinity competitive antagonist for the GIP system with proven activity in humans (81–83).

The GIP receptor (GIPR) belongs to the class B GPCRs, and stimulates the Gα_S_ adenylyl-cyclase-cAMP-PKA pathway. It is expressed in a wide range of tissues and organs, the most important being the endocrine pancreas, adipose tissue, bone, and several CNS regions (71). Accordingly, GIPR signaling has been demonstrated in pancreatic α- and β-cells (84), bone cells (30), adipocytes (85), and hippocampal neural cells (86).The GIP system is less conserved among species compared to the GLP-1 system (87, 88). Thus, the sequence homology of the GIPR between rodents and humans is only 81%, and the GIP peptide has 2 and 3 amino acid substitutions, respectively, in rats and mice compared to human GIP (88, 89). The efficacy of human GIP on rat and mice GIPRs is only 75 and 60%, respectively, of those of rat and mouse GIP, respectively. This information is relevant for *in vivo* and *in vitro* tests of the GIP system in different species (89).

Due to a markedly impaired insulinotropic effect of GIP in T2DM patients (putatively due to a desensitization of the GIP system) (90), the focus for GIP research has changed somewhat from pancreatic β-cell stimulation and glucose homeostasis to other areas, such as metabolism of bones and adipocytes, and neural diseases.

#### GIP's Effect on Bones

The GIPR is expressed in both osteoblast- and osteoclast-derived cell lines (20, 46), and in murine primary cultures of osteoclasts and osteoblasts (33, 34). Aoyama et al. found that the expression of the GIPR increased upon increasing glucose concentrations in the media (46). Expression of the GIPR has, moreover, been verified on human bone marrow-derived mesenchymal stem cells (BMSC) (31).

The osteoblastic cell lines vary in the degree of their maturity, a difference that has been suggested to correlate with GIPR expression. Moreover, the anabolic impact of GIP stimulation on bone parameters such as alkaline phosphatase (ALP), P1NP and cell viability varies between cell lines (20). GIP increases intracellular calcium [Ca^2+^]_i_ and cAMP, and increases expression of P1NP and ALP activity (30). It also increases the expression of c-Fos, an important factor in bone cell proliferation and differentiation (45). Furthermore, GIP improves collagen maturity and fibril diameter in a cAMP dependent manner (91), and stimulates both ALP and mineralization in primary osteoblast cultures from murine BMSC (34). Finally, GIP attenuates caspase 3/7 activity and, thereby, diminishes cell death in both hBMSC and an osteoblastic cell line (31). In primary murine osteoclast cultures, GIP inhibits PTH-induced bone resorption (33). Another study showed that GIP reduces osteoclast formation and resorption in primary human and murine cultures, and this reduced differentiation is independent of the conventional adenylyl-cyclase-cAMP-PKA pathway. GIP decreased the RANKL-induced [Ca^2+^]_i_ increase and calcineurin activity, and decreased nuclear translocation of the RANKL downstream target, NFATc1, which is important for terminal osteoclast differentiation (32).

The first *in vivo* study was conducted in 2001, where Bollag et al. showed a positive impact of native GIP on bone density in ovariectomized (OVX) rats (28). Since then, many studies have been performed using DPP-4 resistant peptides and genetically modified mice with either receptor knock-out (KO), congenital overexpression or deficiency of the GIP peptide. The DPP-4 resistant peptides all show either anabolic or anti-resorptive properties. N-AcGIP, for example, improved cortical bone properties in rats and decreased osteoclast mediated bone resorption in OVX mice, as seen by a reduction in both the number of osteoclasts and the resorption marker CTX (29, 32). In a type 1 diabetes (T1DM) mouse model, short term treatment with D-Ala_2_-GIP, prevented a reduction of bone formation parameters and at tissue level, it improved mechanical properties (36). A peptide hybrid of GIP and oxyntomodulin, stimulating both GIP, GLP-1, and glucagon receptors, also improved cortical bone strength in a T2DM mouse model (92).

Two variants of GIPR KO mice exist, varying in the amount of exons deleted. Both variants have compromised bone properties, but some of the results are conflicting. The first GIPR KO mouse characterized, with deletion of exon 4–5, showed decreased bone formation parameters such as aBMD, bone mineral content (BMC), trabecular bone volume, ALP and osteocalcin, and increased resorptive parameters, such as increased numbers of osteoclasts and increased urinary elimination of the resorption marker, deoxypyridinoline (24, 25). The other GIPR KO mice, with deletion of exon 1-6, showed decreased bone strength and cortical thickness, and increased bone resorption, but they also had an increased number of osteoblasts and a reduced number of osteoclasts (26, 27). The double incretin KO mouse, a combination of a GIPR KO and GLP-1R KO, showed reduction in the cortical properties and a reduced strength of the bones (93). Congenital deficiency of the GIP hormone was similarly consistent with an important role of GIP for bone metabolism, showing decreased bone volume and number of trabeculae, and increased osteoclast surfaces (94). Conversely, overexpression of GIP was associated with increased formation of bone, with an increase in bone mass, number of osteoblasts, osteocalcin levels, and inhibited bone resorption, as indicated by decreased pyridinoline crosslinks and decreased number of osteoclasts (34, 95).

In an early human study evaluating the effects of a brief intravenous injection of GIP, there were no apparent effects on bone resorption (7). A more recent study showed a 50% decrease in CTX upon oral glucose ingestion, and 30% decrease upon intravenous glucose administration, thus with a larger decrease in the presence of high levels of incretin hormones (9). Two subsequent studies showed a robust direct inhibitory effect of GIP infusion on CTX at both low, eu-, and hyperglycemic levels (21), and that the reduction of CTX by GIP was independent of insulin (22). Moreover, a loss-of function GIPR gene polymorphism (E354Q) was correlated with decreased aBMD, as analyzed by DXA-scans in a 10 year follow up study of 1424 perimenopausal women, and an evaluation of registered fractures over a period of 16 years showed a 50% increased fracture risk (23).

Overall, the studies in both humans and rodents indicate GIP to be a pivotal and direct regulator of bone metabolism, with direct anabolic effects on osteoblasts and anti-resorptive effects on osteoclasts.

### Glucagon-Like Peptide 1 (GLP-1)

GLP-1 is encoded within the proglucagon gene, which also codes for glucagon and GLP-2. In the α-cells of the pancreas, the proglucagon peptide is cleaved by prohormone convertase 2 (PC2), yielding glucagon. In the intestinal L-cells, PC1/3 cleaves proglucagon to give the peptides, GLP-1 (PG78-107) (58, 59) and GLP-2. GLP-1 is found in a glycine-extended form, GLP-1 (7-37), which can be C-terminally amidated to give GLP-1 (7-36 NH_2_) (59). It is released primarily in response to nutrient intake, and is less affected by endocrine and neuronal factors. Thus, there is no apparent “cephalic phase” for the meal-induced response (96). As for GIP and GLP-2, GLP-1 is degraded by the enzyme DPP-4 (97), which cleaves it after its alanine in position 2, giving it a half-life <2 min (61, 71, 97–99).

The GLP-1R is a class B GPCR, related to the GIP-, GLP-2- and glucagon receptors and like these, it mainly couples to Gαs (71). Although it is internalized, desensitization may not occur *in vivo* (100). The GLP-1R is found in a variety of tissues, amongst them, the pancreas and CNS, where it regulates release of glucoregulatory hormones and appetite, respectively. GLP-1 acts on the pancreatic β-, α-, and δ-cells, where it stimulates insulin secretion, inhibits glucagon secretion and stimulates somatostatin release, respectively. Moreover, GLP-1R activation in the CNS leads to decreased food intake and weight loss (14), while in the stomach, GLP-1 inhibits gastric motility and acid secretion (71). Due to these beneficial effects of GLP-1R activation on metabolism, several drugs have been developed that act on the GLP-1 receptors, including the GLP-1RAs liraglutide, dulaglutide and exendin-4, used in the treatment of T2DM and obesity (101).

#### GLP-1's Effect on Bones

Several studies indicate that GLP-1 has an effect on bone homeostasis, although the exact mechanism involved remains unclear. The GLP-1R has been found on immature osteoblastic TE-85 and MG63 cells, but not in the Saos2 cell line (20). It was also found in mouse osteoblast-like MC3T3-E1 cells (46, 47), with one of the studies showing that its expression increases with higher glucose concentrations in the media (46). In another study, a GLP-1 receptor distinct from the classical pancreatic-type receptor was found on MC3T3-E1 cells (102). In a study by Meng, the receptor was found in bone marrow stem cells (BMSC). These cells can differentiate into osteoblastic cells, but the GLP-1R has not been found in primary osteoblasts (38), or on the osteocyte-like MLO-Y4 cells (39). Pereira et al. (37) found that the GLP-1R was expressed in mouse bone and bone marrow, and in primary osteoblasts and osteoclasts, and also in the IDG-SW3 osteocytic cell line, but not in the MLO-Y4 osteocytic cell line.

*In vitro* experiments show that activation of the GLP-1R is important in bone metabolism. In one study by Pancheco-Pantoja, GLP-1 increased cell viability and decreased P1NP secretion in two osteoblastic cell lines, TE-85, and MG-63 (20). In another study, they found that GLP-1 induces c-Fos expression (a gene important in bone cell proliferation and differentiation), in osteoblastic TE-85 cells, with a peak induction after 60 min (45). In mouse osteoblast-like MC3T3-E1 cells, the GLP-1RA, exendin-4, increased proliferation, differentiation, and mineralization through a MAPK pathway (47). Likewise, liraglutide, increased proliferation and differentiation in mouse osteoblast-like MC3T3-E1 cells in one study (47, 48), but in another study, where the cells were cultured in a commercial osteogenic differentiation medium, liraglutide inhibited differentiation, as measured by ALP and osteocalcin in both studies (49). In BMSCs, GLP-1 inhibits adipogenic differentiation, while it promotes osteogenic differentiation (38, 40). Pereira et al. found that both exendin-4 and liraglutide increased osteoclast numbers when added to osteoclast precursor cells derived from mouse bone marrow, while addition to mature osteoclasts reduced the resorbed area (39). In addition, GLP-1RAs have been shown to affect osteoclasts, stimulating their differentiation, but reducing the resorbed area (37), and reducing SOST/sclerostin expression in osteocyte-like MLO-Y4 cells (39).

*In vivo*, there are multiple studies in rodents establishing GLP-1's role in bone metabolism. In ovariectomized mice, treatment with exendin-4 and liraglutide both had positive effects on trabecular bone, but no effect on cortical bone. Exendin-4, but not liraglutide, caused an increase in calcitonin, and a decrease in serotonin, while both agonists increased osteoclast differentiation, but reduced the resorbed area (37). In a study in streptotozin-induced T1DM mice, short-term treatment with liraglutide had no effect on bone loss, assessed by micro-CT of trabecular microstructure, or bone formation parameters. It did, however, improve tissue material properties (36). Moreover, both GLP-1R KO and double-incretin receptor KO mice show decreased bone quality and strength and reduced cortical area, as well as decreased collagen maturity (93, 103, 104).

Treatment with GLP-1 in normal, insulin-resistant and type-2 diabetic (T2DM) rats, restored the impaired trabecular structure, and while osteocalcin and osteoprotegerin increased in all three groups, RANKL only increased in the T2DM rats (41). In hyperlipidic rats that displayed osteopenia, short-term treatment with GLP-1 or exendin-4 both reversed the decrease in bone mass and quality, while levels of osteocalcin and osteoprotegerin increased (42). Studies in ovariectomized rats have demonstrated that treatment with the GLP-1RA liraglutide improved trabecular number, volume and thickness, and increased aBMD compared to control rats (40), while exendin-4 treatment increased aBMD and BMC measured by DXA, and improved trabecular structure, assessed by micro-CT, and increased bone strength. Gene analysis also revealed that exendin-4 treatment increased the bone formation markers ALP, osteocalcin, and P1NP, while decreasing the bone resorption parameter CTX (43). Liraglutide treatment of the spontaneously diabetic Goto-Kakizaki rats restored the impaired aBMD, and gene analysis showed an increase in bone formation parameters (44), while treatment with exendin-4 in rats with unloading-induced osteoporosis also improved trabecular structure, aBMD and bone strength (38). Exendin-4 treatment of T2DM OLETF rats also increased femoral aBMD, while reducing sclerostin and increasing osteocalcin (39).

Results from human studies are inconsistent. A randomized controlled study by Iepsen et al. showed that liraglutide had beneficial effects on weight-loss-induced bone loss. After an 8-week weight-loss program, 37 women were divided into a control group (19 women) and a group receiving liraglutide (18 women) in the 52-week weight-maintenance phase. BMC, measured by DXA scan, decreased significantly in the control group, but not in the liraglutide group, whereas P1NP increased significantly in the liraglutide group, but not the control group, indicating that the protective effects are mediated by increased bone formation. There was no effect on bone resorption, measured by plasma CTX (35), which is in accordance with an earlier study by Henriksen et al., where subcutaneous GLP-1 treatment had no effect on CTX in seven healthy participants (7).

As GLP-1 analogs are already in use for the treatment of T2DM, there are meta-analyses looking into the correlation between liraglutide and exenatide treatment and the risk of fractures. Studies on T2DM patients treated with GLP-1R agonists found they were not associated with any change in aBMD (105) and the risk of bone fractures was not altered (106). However, in another meta-analysis of randomized controlled trials, a decreased risk of fragility fractures with liraglutide, but an elevated risk with exenatide treatment was found (107).

In conclusion, multiple studies have demonstrated an impact of GLP-1 on bone metabolism involving both an activation of osteoblast function and an inhibition of osteoclasts.

### PYY

Peptide YY (PYY) is another hormone secreted from the L-cell in the postprandial state. PYY is often co-secreted with GLP-1 and GLP-2 (108) in proportion to caloric intake, and decreases food intake via appetite-inhibiting actions which involve the hypothalamic arcuate nucleus. It belongs to the pancreatic polypeptide family together with neuropeptide Y (NPY) and pancreatic polypeptide (109), and is secreted as the 36-amino-acid molecular form PYY_1−36._ After secretion, PYY_1−36_ is degraded by DPP-4 to form PYY_3−36_ (109).

In general, the different PYY molecular forms have different half-lives and act via the four different G protein-coupled Y receptors to which they bind with different affinities. PYY_1−36_ binds to the Y1, Y2, and Y5 receptors, whereas PYY_3−36_ is highly selective for the Y2 receptor. This leads to opposing effects on appetite and possibly also on glucose homeostasis, as reviewed previously (110). PYY_3−36_ is responsible for the anorexigenic actions of PYY, as documented in infusion studies (111, 112) and together with GLP-1, seems to play a role in the decreased food intake and major weight loss seen after the bariatric surgery procedure, Roux-en-Y gastric bypass (111, 113). Meal-induced PYY secretion is blunted in obese participants, but the anorexigenic effect of PYY seems intact (114). COOH-terminally truncated PYY metabolites, PYY_1/3−34_, have recently been described in humans (115). The biological impact of these metabolites remains to be elucidated, but PYY_1/3−34_ has no affinity for the Y2 receptor (115).

#### PYY's Effect on Bones

PYY may exert catabolic effects on bones. Studies in different populations of patients with different kinds of weight alterations have suggested that changes in PYY plasma concentrations modulate bone homeostasis. Elevated fasting PYY was negatively associated with aBMD in pre-menopausal exercising women (116) and in women with anorexia nervosa (50). A negative correlation between elevated PYY and P1NP in young female athletes with amenorrhea (117) further supports an effect of PYY on bone homeostasis. Likewise, the higher postprandial PYY concentration after Roux-en-Y gastric bypass has been suggested to play a role in the marked bone loss that takes place after surgery, and exceeds what is expected from the major weight loss itself. After gastric bypass, CTX increases and this is directly correlated to the changes seen in PYY (51). In contrast, in patients losing weight after gastric banding, another bariatric procedure, both PYY and CTX concentrations were unchanged, supporting a connection between PYY and bone markers (51).

In addition to these associations in human studies, evidence from rodent studies supports a role for PYY in the regulation of bone homeostasis through modulation of both osteoclast and osteoblast activity. The Y1 receptor has been shown on the osteoblast, and PYY might exert suppressive effects on osteoblast activity via these receptors (53). Accordingly, an overproduction of PYY in transgenic mouse models reduced bone mass, whereas PYY knockout mice displayed increased bone mass and strength (54), although the opposite was shown in a study with another PYY knockout model (55). In addition, selective conditional deletion of hypothalamic Y2 receptors in adult mice led to increased bone volume, indicating that the Y2 receptors may also be involved in bone remodeling (56).

Thus, whereas PYY seems to have robust weight-reducing effects, exogenous PYY administration might have a detrimental effect on the bone density.

## Osteoporosis—Therapeutic Possibilities in the Gut-Bone Axis

Osteoporosis is a bone disease in which the bone becomes more fragile. It represents a growing challenge for health care systems and is also an economic burden, as bone fragility increases the risk of fracture, which is a major cause of morbidity. Fractures often require hospitalization and immobilization, which may cause further complications, and recovery is often slow and incomplete (118, 119). Pathological fractures arise from an imbalance in the bone remodeling process, and are characterized by low bone mass and microarchitectural changes arising from internal factors (primary osteoporosis), such as falling levels of estrogen in postmenopausal women, or external factors (secondary osteoporosis), e.g., malnutrition (120). Pharmacological treatment of primary osteoporosis is divided into two classes of drugs with anti-resorptive and anabolic effects, respectively (121, 122), which is summarized in Table 2.

**Table 2:** Current pharmacological treatment of osteoporosis.

**Type**	**Name**	**Mechanism**
**ANTI-RESORPTIVE**		
Bisphosphonates	Alendronate Ibandronate Risedronate Zolendronic acid	Binds to hydroxyapatite in the extracellular matrix and causes osteoclast cell death by inhibiting the enzyme farnesyl pyrophosphate synthase, disrupting the cytoskeletal structure
Estrogen/SERM	Raloxifene	Binds to the estrogen receptor, which has anti-catabolic effects
Calcitonin		Anti-resorptive effects (in animals)
Antibody mediated	Denosumab	Scavenges RANKL, preventing it from stimulating osteoclast precursor differentiation and maturation
**ANABOLIC**		
PTH-analog (1-34)	Teriparatide	Hormone replacement therapy: intermittent increase in plasma PTH-levels activates osteoblasts. Daily injections
PTH-analog (1-84)	Natpara	Hormone replacement therapy—long term action

*Pharmacological treatment of osteoporosis can be divided into anti-resorptive and anabolic drugs. Bisphosphonates are the most widely used drug for treating osteoporosis. Strontium ranelate is not approved by the FDA, and only restricted use by the EMA, due to severe side-effects, such as increased risk of myocardial infarction and skin reactions. Teriparatide is the only marketed anabolic drug, and mimics endogenous parathyroid hormone (PTH). Intermittent increases in plasma-PTH have anabolic effects, but continuously elevated levels, such as in hyperparathyroidism, have catabolic effects. Several new drugs are being developed, aimed at specific molecules involved in the bone remodeling process, such as inhibitors of cathepsin K (an enzyme secreted from osteoclasts, necessary for the resorption process) and inhibitors of the Wnt/β-catenin-pathway inhibitors sclerostin and dickkopf-1*.

Due to lack of efficiency and intolerable side-effects, current treatment of osteoporosis is limited, and several new drugs are being developed, targeting specific molecules important for bone homeostasis. Examples hereof are inhibitors of sclerostin and dickkopf-1, which are inhibitors of the Wnt signaling pathway, and inhibitors of cathepsin K, which is secreted from osteoclasts and important for the resorption process (121, 122).

The strategy of using GLP-2 for the treatment of osteoporosis has been pursued previously resulting in a series of human studies investigating acute and chronic effects of daily GLP-2 injections on bone remodeling (7, 17–19, 76–78, 123). However, despite a strong inhibition of osteoclast activity and a significant increase in aBMD after 4 months treatment (17), GLP-2 has not reached the market as a new drug for the treatment of osteoporosis, although a GLP-2 analog (teduglutide) was approved by FDA in 2012 for the treatment of SBS because of its beneficial effects on intestinal function (63). As there are already drugs on the market based on GLP-1, the effects on bone metabolism may expand the use of these drugs, or aid the development of drugs more specifically targeted at bone metabolism. GLP-1 based drugs have anorexic effects through their activity in the hypothalamus, which may limit their efficacy as anti-osteoporosis therapy since food intake plays a pivotal role in the maintenance of strong bones. Another potential strategy in the development of drugs based on the hormones in the gut-bone axis, is to target not one, but two or more receptors. This multi-agonism approach may have synergistic effects, and there are studies showing synergistic effects on the treatment of metabolic diseases, such as T2DM (92, 124, 125). Moreover, one study has shown that a GIP-oxyntomodulin hybrid peptide (targeting GIP, GLP-1 and glucagon receptors) had beneficial effects on bone loss in db/db mice with T2DM (36). As all the discussed hormones have anabolic and/or anti-catabolic effects on bone metabolism, all of their cognate receptors are of interest.

**Figure 2:**
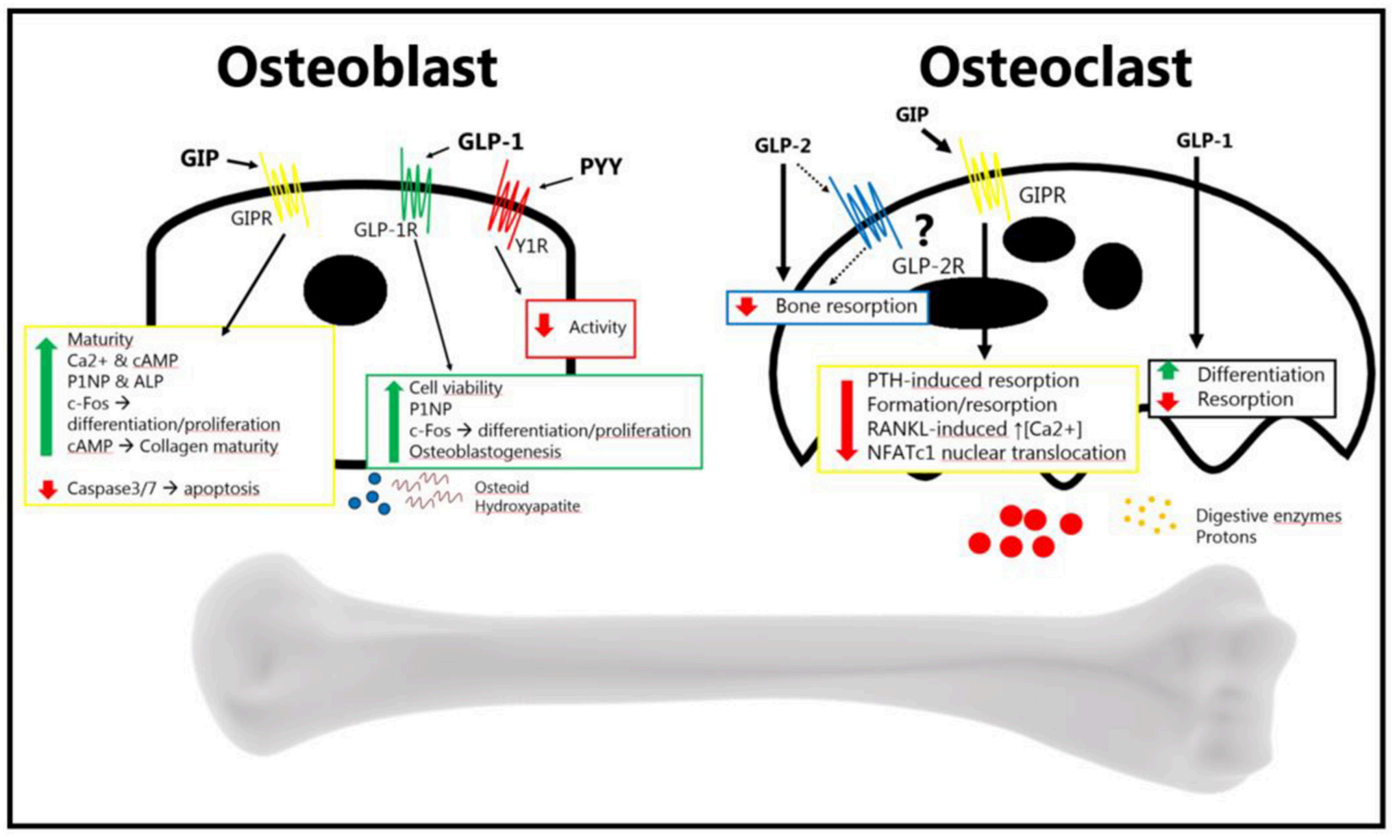
The gut hormones GLP-2, GIP, GLP-1, and PYY, have been shown to affect bone metabolism. The osteoblast increases its activity in response to GIP and GLP-2 (anabolic effects), and decreases its activity in response to PYY (catabolic effects). The exact mechanism of GLP-2 remains to be elucidated. GLP-2 has been shown to be anti-resorptive *in vivo*, an effect which may be direct or indirect. GIP decreases osteoblast activity, and GLP-1 also seems to decrease resorption. PYY's effect, if there is one, has yet to be determined. GLP-2 has been shown to decrease bone resorption, but it is uncertain whether it affects the osteoclast directly. GIP has been shown to affect the osteoclast, reducing bone resorption. This has also been shown for GLP-1, while it has also been shown that it increases differentiation. There is no certain effect of PYY on osteoclasts (Bone figure from Somersault18:24, CCBY-NC-SA 4.0).

In summary, the gut is an important regulator of bone homeostasis, with several gut-derived factors controlling bone formation and resorption (Figure 2). The current treatment of osteoporosis is limited, and as GPCRs in general are excellent drug targets, it will be exciting to follow whether novel drugs targeting gut hormone receptors will in the future, reach the market for the treatment of osteoporosis.

## Author Contributions

SS, KS-J, JW, and MS wrote initial drafts of selected parts of the manuscript. All authors contributed to the writing of the manuscript. MR, BH, and JH assembled, reviewed, and corrected the manuscript.

### Conflict of Interest Statement

MR, BH, and JH are co-founders of Bainan Biotech, focusing on novel treatment of osteoporosis. The remaining authors declare that the research was conducted in the absence of any commercial or financial relationships that could be construed as a potential conflict of interest.
